# Are Vegetative Reproduction Capacities the Cause of Widespread Invasion of Eurasian Salicaceae in Patagonian River Landscapes?

**DOI:** 10.1371/journal.pone.0050652

**Published:** 2012-12-04

**Authors:** Lisa K. Thomas, Lena Tölle, Birgit Ziegenhagen, Ilona Leyer

**Affiliations:** 1 Department of Conservation Biology, Faculty of Biology, Philipps-University Marburg, Marburg, Germany; 2 Department of Geisenheim, Plant Ecology and Nature Conservation, University of Applied Sciences, Hochschule RheinMain, Geisenheim, Germany; Jyväskylä University, Finland

## Abstract

In recent decades, invasive willows and poplars (Salicaceae) have built dense floodplain forests along most of the rivers in Patagonia, Argentina. These invasion processes may affect *Salix humboldtiana* as the only native floodplain tree species in this region. It is assumed, that the property to reproduce vegetatively can play an important role in the establishment of invasive species in their new range. Thus, in order to contribute to a better understanding of willow and poplar invasions in riparian systems and to assess the potential impacts on *S. humboldtiana* the vegetative reproduction capacities of native and invasive Salicaceae were analysed. In a greenhouse experiment, we studied cutting survival and growth performance of the three most dominant invasive Salicaceae of the Patagonian Río Negro region (two *Salix* hybrids and *Populus* spec.), as well as *S. humboldtiana,* taking into account three different moisture and two different soil conditions. In a subsequent experiment, the shoot and root biomass of cuttings from the former experiment were removed and the bare cuttings were replanted to test their ability to re-sprout. The two invasive willow hybrids performed much better than *S. humboldtiana* and *Populus* spec. under all treatment combinations and tended to re-sprout more successfully after repeated biomass loss. Taking into account the ecology of vegetative and generative recruits of floodplain willows, the results indicate that the more vigorous vegetative reproduction capacity can be a crucial property for the success of invasive willow hybrids in Patagonia being a potential threat for *S. humboldtiana*.

## Introduction

The introduction of exotic species is one of the most serious causes of man-made changes in ecosystems and grew with increasing human migration and expanding trade [1]. When they naturalize and expand their range, introduced non-native species are called invasive [2] and can threaten ecosystems, habitats or species with their establishment and spread. Floodplains are known to be very sensitive to plant invasion because of regular natural as well as human-induced disturbances [3], [4]. The hydrological connectivity of river corridors facilitates the dispersal of introduced invasive organisms [5] and the bare-ground sites arising after flood events are ideal for the establishment of invasive pioneer species [6]. In this context, willows and poplars are very successful in occupying new riparian habitats. In the southern hemisphere, introduced willows have widely naturalized along river margins including South Africa, Australia, New Zealand and South America [7]–[10]. In Australia, some invasive *Salix* species were declared as ‘weeds of national significance’ with e.g. *S. cinerea, S. babylonica* and *S. fragilis* spreading aggressively [11], [8], [12]. Salicaceae are known to alter fluvial dynamics and to facilitate the development and growth of sand bars and islands [13] and thus have traits that could alter the ecosystem profoundly if they are invasive. Other possible consequences of invasive willows are the displacement of native vegetation resulting in a loss in biodiversity, the obstruction and diversion of streams and consequent erosion [8]. Reduction in the quantity and quality of water and changes in light conditions have consequences for e.g. macroinvertebrates [14] and bird assemblages [15].

Floodplain willows reproduce sexually as well as asexually. Sexual reproduction occurs through the release of a large number of minute, light seeds, which are readily dispersed by wind and water [13]. Seedling survival and establishment is controlled by various factors such as the presence of bare-ground sites and sufficient soil moisture. They do not tolerate burial by sedimentation nor extended submersion by flooding during the growing season [16]. Therefore, in unfavourable years, sexual reproduction can be a rare event [17]. The ability to reproduce vegetatively is frequently linked to species with a high risk of becoming invasive [18]. Vegetative reproduction provides an alternative regeneration strategy that is less sensitive in its environmental requirements due to carbohydrate reserves and pre-formed root and shoot primordia [19]. Generally, whenever fragments of branches fall into the water and are swept away by the river, they are able to re-sprout as soon as they are deposited on suitable habitats [20]. Since regeneration from seeds needs favourable conditions in a very short time period, vegetative fragments play the major role for *Salix* distribution after flood events [21]. Indeed, some studies already demonstrated the importance of vegetative reproduction for the invasive spread of willows (e.g. [22]–[25]).

In Northern Patagonia, invasive willows and poplars of Eurasian origin have built floodplain forests in almost all riparian landscapes. Mainly willows originating from Europe dominate the Patagonian rivers [26], [25]. Probably introduced to South America by European settlers in the 19^th^ or 20^th^ century, their distribution area has increased significantly in the last two decades [25]. One of the largest rivers in this region is the Río Negro, where dense gallery forests occur which are mainly composed of two invasive willow hybrids as well as poplar hybrids. The only native tree species in this area, *Salix humboldtiana,* is distributed throughout the warm regions of South America [27] but has become relictual in some places due to competition with invasive species [28]. The question arises as to whether *S. humboldtiana* can keep up with the invasive Salicaceae or whether it has been affected or will be affected by the still ongoing invasion process.

The aim of the study was to assess whether and how vegetative reproduction as one part of the life cycle could play a prominent role for the spread and establishment of invasive willows in Patagonian river landscapes and therefore contribute to a better understanding of *Salix* invasions in riparian systems.

Consequently, the following questions were addressed in two greenhouse experiments:

- Do the invasive Salicaceae perform better than the native one regarding survival and growth performance of cuttings taking into account soil moisture and soil composition gradients which correspond to field conditions?
- Do invasive Salicaceae possess better re-sprouting capacities and perform better than *S. humboldtiana* after repeated shoot and root removal?

## Methods

### Ethics Statement

No specific permits were required for the described sampling in the study area. The locations where branches were collected are not privately-owned or protected in any way. Field studies did not involve endangered or protected species.

### Study Species and Sampling Location

The natural vegetation of the Río Negro floodplain is composed of *Salix humboldtiana* as the only native tree species and shrubby vegetation (e.g. *Larrea spp., Prosopis spp., Baccharis spp.*) [29]. Introduced tree and shrub species propagate intensively, mainly willows (*Salix spp.),* poplars (*Populus spp.),* salt cedar *(Tamarix spp.)* and Russian Olive (*Eleagnus sp*.). The Río Negro is located in the shrubby Monte steppe in northern Patagonia, Argentina. The climate is arid with a mean annual precipitation of less than 250 mm and a mean annual temperature of 14–20°C [29]. The Río Negro is formed by the confluence of the Limay River and the Neuquen River and discharges into the Atlantic Ocean. It is about 600 km long and has a mean annual discharge of about 900 m^3^/s with pronounced water level fluctuations (DPA, Departamento Provincial de Aguas Río Negro). Both feeder rivers have several dams for energy generation purposes while the Río Negro itself is not regulated by dams and is characterised by pronounced erosion and sedimentation processes. However, unseasonal flooding events due to the dams in the feeder rivers influence the Río Negro as well.

For the greenhouse experiments, *S. humboldtiana* and the three most common invasive Salicaceae were chosen. These are *S. x rubens* (hybrid between *S. alba* and *S. fragilis*), a hybrid of *S. babylonica* and taxa of the *S. alba* - *S. fragilis* hybrid swarm including the parent taxa (hereafter referred to as *S. babylonica* hybrid), as well as *Populus* spec. which probably consists of different hybrids with *P. nigra var. italica* and other invasive *Populus* taxa involved as parents (e.g. *P. deltoides*, *P. x canadensis*). The determination of *S. humboldtiana* and *Populus* spec. could be carried out without difficulty due to characteristic leaf forms. Distinguishing features of the *S. babylonica* hybrid in comparison to *S. x rubens* are the earlier leaf development in spring as well as its hanging branches. In a preliminary study several samples were genetically determined using microsatellite markers sb243, sb194 [30] and sb880 [31] to relate the morphological characteristics of the hybrids to the taxonomical status (Mengel, C. pers. comm.). It could be shown, that especially the earlier leaf development of the *S. babylonica* hybrid could be used for determination purposes. For each target species, 30 cuttings were harvested from twigs from each of 10 randomly chosen adult individuals. As minimum distance between individuals of the same species 100 m were defined to avoid sampling shoots of the same mother tree. The diameter and length of the samples collected ranged between 5 and 15 mm and between 20 and 26 cm, respectively, according to literature recommendations (e.g. [32]). The twigs were cut in February 2010 at the Negro River near General Roca (39°06′ S, 67°37′ W) at the upper reach of the river and Luis Beltrán (39°15′ S, 65°45′ W) at the middle reach. The well-developed shoots with leaves were removed before the cuttings were wrapped in moist tissues and sent to Germany, where they were stored for two weeks in darkness at 4°C. Some cuttings across all taxa started to re-sprout slightly or develop roots during storage. Those sprouts and roots were cut off the day before the experiment started.

**Figure 1:**
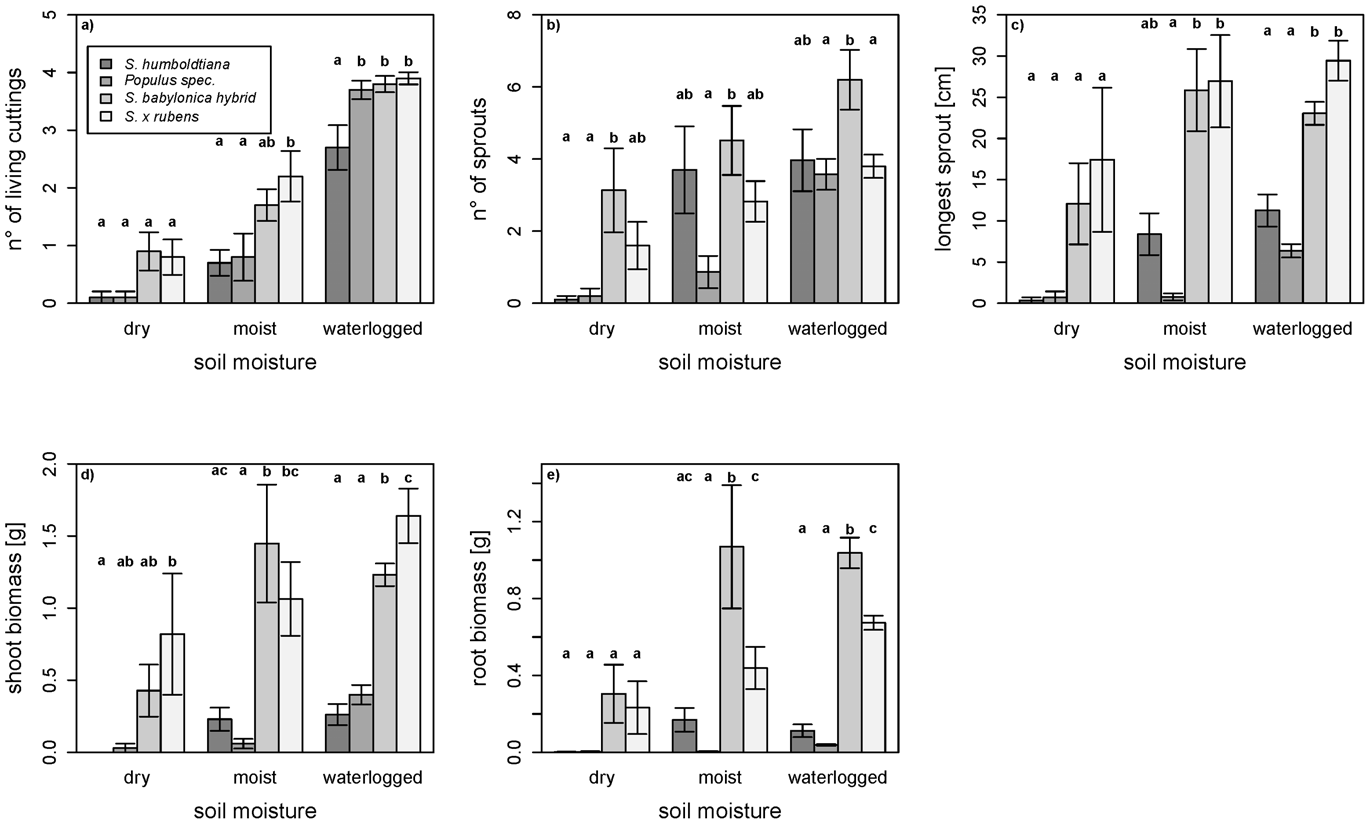
Effects of species and soil moisture. Effects of species and soil moisture on a) number of living cuttings, b) number of sprouts, c) longest sprout, d) shoot biomass and e) root biomass (means±SE) after 84 days. Different letters indicate Tukey’s HSD between the species (p<0.05).

**Table 1:** Main effects of species, soil moisture and soil composition on the survival rate (living cuttings) and growth performance (n° of sprouts, longest sprouts, shoot biomass, root biomass) after 84 days (three-way ANOVAs; response variables were square-root-transformed to improve the diagnostic plots) and general linear mixed models of the number of sprouts and the longest sprout over time.

	D.F.	Living cuttings(ANOVA)	N° of sprouts(ANOVA)	Longest sprouts(ANOVA)	Shoot biomass (ANOVA)	Root biomass (ANOVA)	N° of sprouts(GLMM)	Longest sprout(GLMM)
Species	3	10.42 ***	9.24***	21.39***	26.52***	37.80***	9.69***	10.64***
Moisture	2	93.0 ***	31.35***	20.48***	24.20***	22.84***	25.48***	52.75***
Soil	1	3.52	4.40*	5.38*	5.40*	0.15	4.32*	0.10
Species: Moisture	6	1.28	0.28	0.76	1.14	2.21*	1.46	7.19***
Species: Soil	3	0.56	0.90	0.74	1.18	0.40	0.37	1.58
Moisture: Soil	2	4.90 **	0.25	0.96	0.95	0.41	1.18	0.07
Species: Moisture: Soil	6	1.21	0.63	0.38	0.39	0.97	0.74	0.88
Residuals	96							

Values are F-values. Levels of significance (p) are denoted with *p<0.05, **p<0.01 and ***p<0.001.

### Experimental Design

#### Performance experiment

The cuttings of the four taxa involved were subject to three water and two soil treatments in a full factorial design. Two substrate combinations were chosen that imitate the natural conditions in Patagonia resulting from 164 analysed soil samples in the field. For fine-grained soil, a mixture of 40% sand, 50% loam and 10% humus was used and for coarse-grained soil, a mixture of 60% gravel, 30% sand, 5% loam and 5% humus. The soil were filled in perforated PVC boxes (30 cm * 20 cm * 5 cm) and these were inserted into trays to apply three water level treatments. These treatments covered a wide range of the large moisture gradient along which floodplain willows and poplars can grow. We included the treatments ‘dry’ (trays filled with water up to 1 cm after it had been dried out), ‘moist’ (water level permanently app. 2 cm under soil surface) and ‘waterlogged’ (cuttings permanently covered with water). Each factor combination was replicated five times for each taxon. Four bare cuttings of different individuals of the same taxon were laid horizontally in one box resulting in 30 boxes and 120 cuttings per taxon. It was ensured that four different individuals per taxon were chosen for each box by randomly selecting four cuttings from four of the ten ‘mother’ trees. Between 10 and 14 cuttings per individual were used in the experiment. Five response variables were measured: (i) number of living cuttings as a measure for cutting survival, (ii) number of sprouts, (iii) length of longest sprout emerging from a cutting as well as (iv) shoot and (v) root biomass. Cuttings were defined as dead when they did not develop sprouts until the end of the study. Except for the number of living cuttings which were noted as absolute numbers per box, analyses were conducted box wise as average value of each response variable of living cuttings per box. Therefore, the statistical replicates for all response variables were boxes, not the number of cuttings in each box. The multi-measured variables (i-iii) were taken five and four times within twelve weeks. After the last measurement on day 84, the shoot and root biomass of each cutting was harvested, oven dried in paper bags for one day at 90°C and weighed.

To avoid local effects, all boxes were located randomly in the greenhouse and shuffled every eight to eleven days. During the whole greenhouse experiment, temperature and light conditions remained constant at 15–20°C and 12 hours a day corresponding to the temperature at the Río Negro in spring. Cuttings were treated twice with nematodes (*Steinernema felitidiae*) since large numbers of fungus gnats appeared in the greenhouse. To avoid fungi infection (Ascomycetes), fungicides were applied every ten days (Universal-Pilzfrei M® and Duaxo Universal Pilz-frei®). Additionally, a biological broad-band insecticide (Spruzit®) was used once.

#### Biomass removal experiment

In a second experimental regime, the re-sprouting capacity after a further shoot and root biomass loss was tested in order to gain insight into the regeneration potential of the different taxa after multiple disturbances. For this purpose, cuttings of the four taxa subjected to the first experiment were used again after the shoot and root biomass harvest. Since an insufficient number of cuttings survived in the former ‘dry’ treatment, only cuttings of the moist and waterlogged water level treatments were included. Cuttings of the four taxa were chosen randomly from different individuals and each planted in a single box (275*88*65 mm) under standardised moisture and soil conditions (waterlogged; soil mixture of 30% gravel, 30% sand, 30% loam and 10% humus). Each factor combination of the previously applied treatments was replicated five times, summing up to a total of 20 cuttings per taxon. All boxes were located randomly and shuffled every seven days. The response variables (i) alive (ii) number of sprouts and (iii) length of longest sprout were measured once after 40 days.

**Figure 2:**
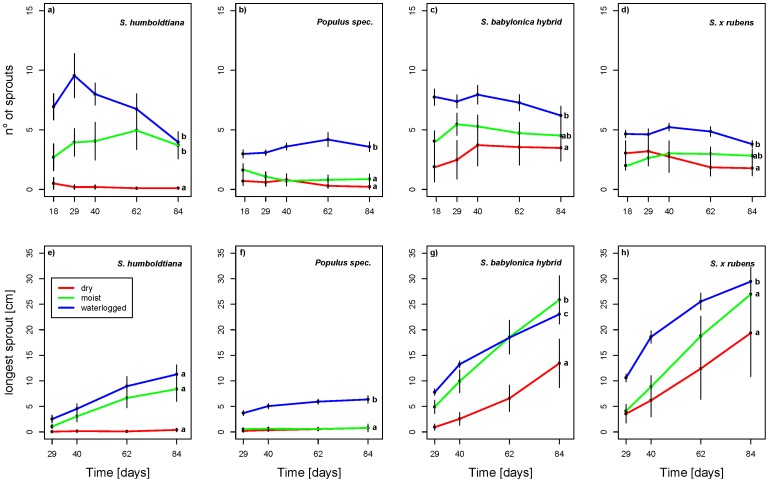
(a–h) Growth performance over time. Growth performance over time of the four species investigated under different soil moisture conditions. (a–d): effects on the number of sprouts (means±SE) after 18, 29, 40, 62 and 84 days; (e–h): effects on the longest sprout (means±SE) after 29, 40, 62 and 84 days. Different letters indicate differences (p<0.05) after Tukey’s all-pairwise comparisons.

### Data Analysis

In the performance experiment, differences among taxa, soil moisture and soil composition concerning the five response variables were tested with three-way factorial ANOVAs followed by Tukey’s method of honestly significant differences.

To test the growth performance over time (number of sprouts, length of the longest sprout), general linear mixed models (GLMM) [33] were used with the repeated measures as the random factor.

Data analysis was carried out using the software package R (Version 2.12.2, R Development Core Team 2011). For the GLMM, the *lme* function in the software package *nlme* [33] was applied. Tukey’s all-pairwise comparisons were conducted in R with the *glht* function in the software package *multcomp* [34].

In the biomass removal experiment, the re-sprouting capacity after shoot and root biomass removal was compared pairwise among taxa with binominal proportion tests [33]. The effects of the taxa and the previously applied treatments (soil moisture and soil composition) on the response variables number of sprouts and length of longest sprout were tested with a three-way ANOVA followed by Tukey’s post-hoc test.

Constancy of variance and normality of errors were checked using diagnostic plots. If not fulfilled, the response variables were square root- or log-transformed [33].

## Results

### Performance Experiment

After 84 days, the *S. babylonica* hybrid (53.3%) and *S. x rubens* (57.5%), showed a higher survival rate than *S. humboldtiana* (29.2%) and *Populus* (38.3%). Species and moisture had profound effects on the number of living cuttings, the number of sprouts and the length of the longest sprout while the soil composition had lesser effects (Table 1). Generally, the growth performance increased for all taxa from dry to moist and waterlogged conditions, and for all treatments the invasive willows achieved the best results (Fig. 1). Independently of the taxa, a better performance could be noted in the fine than in the coarse-grained soil for some response variables (number of sprouts, length of longest sprout, shoot biomass; Table 1).

**Figure 3:**
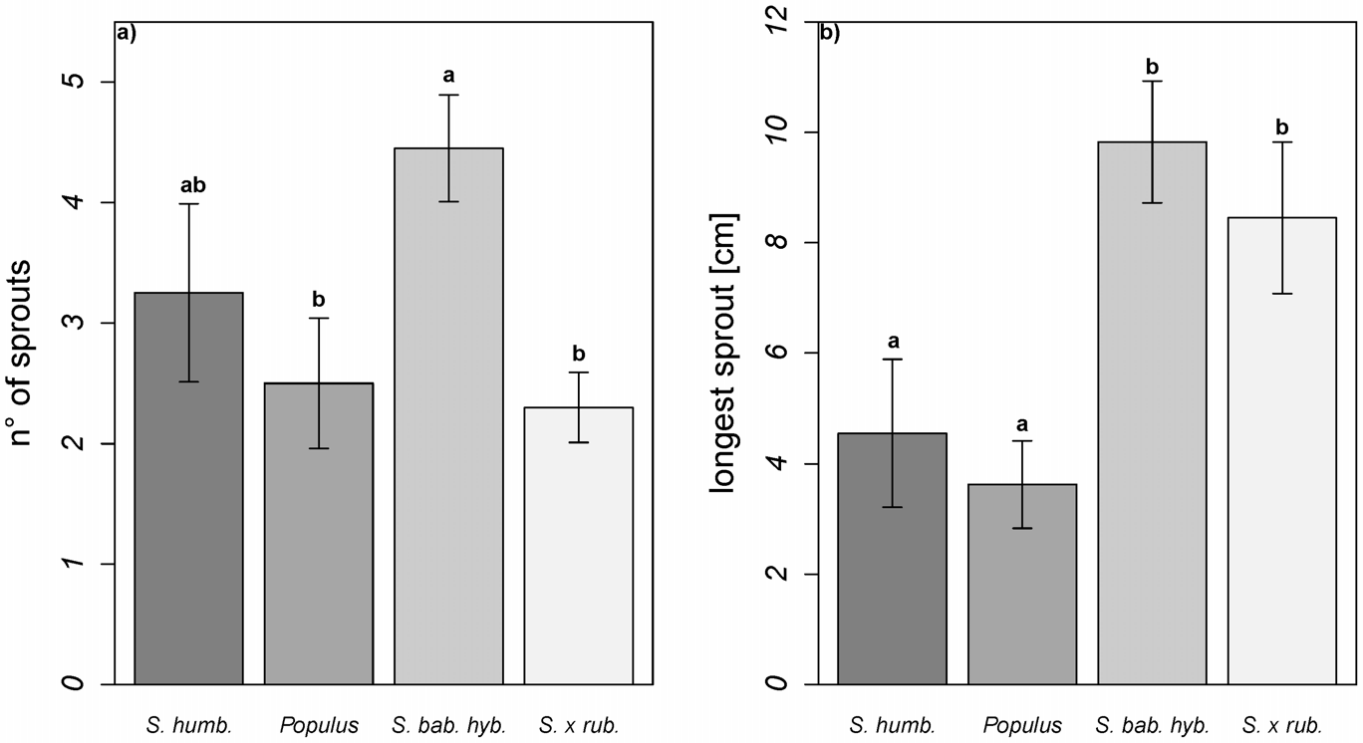
(a,b) Secondary re-sprouting capacities. Secondary re-sprouting capacities measured as a) number of sprouts and b) longest sprout of the four species after 40 days (means±SE). Different letters indicate Tukey’s HSD between the species (p<0.05). *S. humb.* – *Salix humboldtiana*, *Populus* – *Populus* spec., *S. bab.* hyb. – *Salix babylonica* hybrid, *S. x rub*. – *Salix x rubens*.

**Table 2:** Main effects of species, soil moisture and soil composition on the secondary re-sprouting capacities (number of sprouts and longest sprout) in the 2^nd^ experiment after 40 days.

	D.F.	N° of sprouts	Longest sprout
Species	3	4.62**	9.82***
Moisture	1	6.48*	5.06*
Soil	1	4.71*	2.36
Spec: Moisture	3	1.18	1.44
Spec: Soil	3	1.03	0.89
Moisture: Soil	1	0.27	0.00
Spec: Moisture: Soil	3	2.35	0.89
Residuals	64		

Response variables were log-transformed to improve the diagnostic plots. Values are F-values of three-way ANOVAs. Levels of significance (p) are denoted with *p<0.05,**p<0.01 and ***p<0.001.

Overall, the cutting survival tended to be lower for *S. humboldtiana* and *Populus* than for the two invasive willows with some significant results under moist and waterlogged conditions. Under dry conditions, almost all cuttings of *S. humboldtiana* and *Populus* died off (Fig. 1). For the number of living cuttings, an interaction between soil moisture and soil composition could be observed (Table 1) which was visible in the moist water treatment with a higher survival in the fine soil composition than in the coarse one. Regarding the number of sprouts, the *S. babylonica* hybrid performed best under all moisture conditions and, except under dry conditions, *S. humboldtiana* developed more or a similar number of sprouts in comparison to *Populus* and *S.* x *rubens* (Fig. 1). Additionally, in the waterlogged treatment, *S. humboldtiana* showed the most profound decline in the number of sprouts over time (Fig. 2 a) while *Populus* had the fewest sprouts among all taxa (Fig. 2 b). With respect to the lengths of the longest sprout, the two invasive willows performed much better than *S. humboldtiana* and *Populus* (Fig. 1) with a steep rise over time (Table 1, Fig. 2 e–h). In accordance with these results, the two invasive willows performed considerably better than *S. humboldtiana* and *Populus* concerning shoot and root biomass production (Fig. 1 d,e).

### Biomass Removal Experiment

Re-sprouting proportions after further shoot and root removal tended to be higher in the two invasive willows (*S. babylonica* hybrid: 100%; *S. x rubens* 90%) in comparison to *S. humboldtiana* (75%) and *Populus* (70%), which was significant between *S. babylonica* hybrid and *Populus* (Χ^2^  = 4.90; df  = 1; p<0.05) and exhibited a clear trend in the case of *S. babylonica hybrid* and *S.* *humboldtiana* (Χ^2^  = 3.66; df  = 1, p = 0.056). The number of sprouts and longest sprout depended significantly on species and to a lesser extent, on the previously applied water treatment (Table 2). The *S. babylonica* hybrid produced the highest number of sprouts and differed significantly from *S.* x *rubens* and *Populus*. The two invasive willows had significantly longer sprouts than *S. humboldtiana* and *Populus* (Fig. 3). The former soil composition had slight effects on the number of sprouts with more sprouts emerging from cuttings which previously grew on fine grained soil (Table 2).

## Discussion

While all studied taxa were able to reproduce vegetatively, the two invasive *Salix* hybrids clearly out-performed the native *S. humboldtiana* as well as the studied *Populus* species. The questions arise as to how important are the better vegetative reproduction capacities of invasive riparian willows for the ongoing invasion process and whether an out-performance may lead to an out-competing of the native *S. humboldtiana*.

In this context, both regeneration strategies, sexual vs. asexual, have to be taken into consideration. Field observations in the study area revealed that *S. humboldtiana* as well as the *Populus* taxa produce a huge amount of seeds while capsules of the invasive willows contain few or no seeds (personal observation). Simultaneously, vegetative reproduction by broken twigs was observed in all taxa with *S. x rubens* having a great advantage due to the considerable brittleness of the twigs [20], [25]. These findings suggest profound differences in reproduction strategies among *S. humboldtiana* and *Populus* on the one hand and the invasive willows on the other. Since a considerable number of bare-ground sites suitable for germination are recurrently created by hydrogeomorphologic processes, at first glance, sexual reproduction appears to be a successful strategy to establish self-sustaining *S. humboldtiana* as well as *Populus* stands. However, it is reported that broken twigs of Salicaceae can tolerate broader environmental conditions than their seedlings and usually have higher survival rates. Seedlings are well known to be more susceptible to unfavorable growing conditions such as the mechanical impacts of floods, burial and drought stress as compared to vegetative propagules [35], [13]. Additionally, the establishment of vegetative fragments is not restricted to short time periods such as seedling establishment, which is only possible during the seed-release period. The very short life span of the seeds ranging from several days to few weeks [16], further restricts successful sexual reproduction. Therefore, in unfavourable years, this process can be a rare event even under natural conditions [17] and it can be assumed that under unseasonal flood disturbances, e.g. due to river regulation by dams, the property to establish successfully by vegetative propagation could be a great advantage. Indeed, the intensity of alien willow spreading obviously increased after the onset of river regulation in the feeder rivers in the 70 s of the last century, as reported consistently by residents of the Río Negro region.

Regarding the responses of the four studied taxa in our experiment, due to their vigorous growth, the longer sprouts of the invasive willows may contribute to a more successful establishment in comparison to *S. humboldtiana* and *Populus.* This property offers the chance of fragment survival even under competition conditions and even under flood events by escaping from shade, being buried and being under water [36]. With respect to water availability, the invasive willow cuttings performed much better under both drought and sufficient moisture availability, indicating the tolerance to a rather broad range of moisture conditions. In contrast, drought periods probably affect the other two studied taxa to a greater extent. The reason may be that they rely more on sexual reproduction which is per se drought-sensitive. Thus, the poor vegetative reproduction capacity under drought conditions indicates that they cannot compensate for a drought-induced failure in sexual reproduction.

A potential further reason for the invasion success of Eurasian riparian willows at the Río Negro could be that the abundance of *S. humboldtiana* is restricted at this range edge due to unfavourable growing conditions. Its distribution centre is located in the tropical parts of South America, where it forms monospecific stands and flowers the whole year around. Vegetative reproduction occurs in the tropics as well [37], [27]. At the edge of its distribution area a naturally lower abundance and inferior competitive vigour could probably make *S. humboldtiana* more susceptible to competing invasive species in Patagonia than in other regions. However, recent reviews draw the conclusion that the abundance centre hypothesis with decreased abundance and fitness at range edges can not be considered as a general rule (e.g. [38], [39]). Further research is necessary, including the tropical centres as well as other range edges of *S. humboldtiana* (e.g. at Mexican rivers as the northern edge) to shed light on this topic.

It needs to be mentioned that extended vegetative reproduction can be accompanied by low clonal diversity or even monoclonality of stands. This harbours a potential disadvantage since it can affect the adaptation potential under changing environmental conditions [40]. While scenarios of monoclonality have been found at other Patagonian rivers, interestingly, different clones of *S. x rubens* were identified at the Río Negro [25]. Moreover, both sexes as well as viable seeds, although not frequent, were observed for the invasive willow taxa in our study area. Therefore, it cannot be excluded that potential negative impacts of low clonal diversity can be overcome in the future.

Finally, it should be considered that *S. humboldtiana* is known to form hybrids with the studied willow complexes in controlled crosses [41], [42]. Since flowering periods are overlapping (personal observation), natural hybridization processes between native and invasive willows are possible which could lead to introgressive gene flow as e.g. Ziegenhagen et al. [43] demonstrated for *Populus nigra* in its European range.

In conclusion, our data gathered through a greenhouse study indicates that the vegetative reproduction capacities could foster the success of invasive willow hybrids along the Río Negro. However, further research is needed to test if the non-native species may outcompete and impact the native under field conditions.
